# Disseminated intravascular coagulation type of amniotic fluid embolism: a challenging case report with favorable outcome

**DOI:** 10.11604/pamj.2021.38.325.23434

**Published:** 2021-04-01

**Authors:** Mouna Gara, Arij Draouil, Ahmed Ben Saad, Manel Njima, Asma Ladib, Ons Cherif, Ali Jlali, Lotfi Grati

**Affiliations:** 1 Anesthesia-Intensive Care Department B, Maternity and Neonatology Teaching Center of Monastir, Monastir, Tunisia,; 2 Pulmonology Department, Fattouma Bourguiba Teaching Hospital, Monastir, Tunisia,; 3 Department of Pathology, Fattouma Bourguiba Teaching Hospital, Monastir, Tunisia,; 4 Department of Gynaecology-Obstetrics, Maternity and Neonatology Teaching Center of Monastir, Monastir, Tunisia

**Keywords:** Embolism, amniotic fluid, obstetric delivery, prognosis, case report

## Abstract

Amniotic fluid embolism (AFE) is an unforeseeable, life-threatening complication of pregnancy and child birth. Although rare in an absolute sense, most contemporary series of maternal deaths from developed countries report AFE as a leading cause of mortality in the pregnant population. It has a heterogeneous presentation. This clinical heterogeneity makes the diagnosis of AFE difficult based on a beam of clinical and para-clinical arguments. Rapid diagnosis and immediate interdisciplinary treatment are essential for a good outcome. The present is a case of AFE with a disseminated intravascular coagulation (DIC) and a cardiorespiratory collapse following a vaginal delivery.

## Introduction

Despite its low incidence (only 2-8 of every 100,000 deliveries) [1], amniotic fluid embolism (AFE) is considered among the leading direct causes of maternal death with a significant maternal and perinatal mortality estimated to be between 11 and 44% of all maternal deaths [1]. Previous studies revealed mortality rates as high as 61-86%. This decrease in risk for maternal mortality from AFE may be the result of early diagnosis and better resuscitative care as well as changes in case inclusion criteria [1, 2]. Reported risk factors for amniotic fluid embolism include situations in which the exchange of fluids between the maternal and fetal compartments is affected, such as during operative delivery (cesarean or vaginal), placenta Previa, placenta accreta, and abruption [2]. Other possible risk factors include cervical lacerations, uterine rupture, eclampsia, polyhydramnios, and multiple gestations [3]. The main clinical features of AFE are severe hypotension, arrhythmia, cardiac arrest, pulmonary and neurological manifestations, and profuse bleeding caused by disseminated intravascular coagulation. Diagnosis of AFE is based on clinical symptoms after excluding other causes/diagnoses. Its treatment requires immediate, optimal interdisciplinary cooperation, to ensure the airway security, the adequate oxygenation, circulatory support, and correction of haemostatic disturbances.

## Patient and observation

A 33-year-old gravida 3; para 2 woman presented at 39 weeks gestation to the prenatal care unit in labor after an uneventful pregnancy. She was otherwise healthy, she was not taking any medications. The initial examination noted a cervix dilated up to 3cm, the amniotic sac was intact and it was artificially ruptured as the labor progressed. Laboratory findings on admission were normal (hemoglobin: 14.3g/dl; platelets: 252000/mm^3^; prothrombin: 88%). The delivery of a eutrophic female was normal with an Apgar score of 9. Two hours after the delivery, an excessive vaginal bleeding was noted. Hence a manual examination of the uterine cavity was rapidly performed and the patient was giving 5UI of oxytocin and 2g of intravenous Ampicillin. Shortly after, the patient presented a generalized tonic-clonic seizure that lasted about 5 minutes and had spontaneously ceased. At this stage our team of reanimation intervened, upon the initial assessment the patient was unconscious, a cutaneous rash was noted in her lower limbs. Cardio-respiratory auscultation didn´t show any abnormalities and the capillary blood glucose was up to 1.87g. The patient received 1mg of clonazepam and 200mg of hydrocortisone hemisuccinate and was immediately transferred to the operating room for stabilization. Ten minutes after, the patient´s heart beat decreased profoundly to 38 beats/min and no blood pressure could be measured. Immediate cardiopulmonary resuscitation was initiated. The patient was intubated and received 1mg of atropine, 1mg of adrenaline: a normal heart beat was restored and the blood pressure was maintained within the normal range by noradrenalin continuous infusion of 3.5mg/h. the patient was transferred to the intensive care unit (ICU). The plausible diagnoses at this first stage were an anaphylactoid chock, eclampsia and the amniotic fluid embolism.

Laboratory findings on admission showed elements of multi-organ dysfunction: metabolic acidosis with high level of lactates (pH: 7.03; HCO3: 12.4mmol/L; PaCO2: 47mmHg; lactates: 7.6mmol/l), acute renal deficiency (blood creatinine level increased from 70 to 161µmol/L), hepatic cytolysis (aspartate aminotransferases (ASAT): 188UI/L/alanine aminotransferases (ALAT): 62UI/L), Rhabdomyolysis (lactate deshydrogenase (LDH): 1171UI/L) and elements of disseminated intra-vascular coagulation (prothrombin: 37%; activated clotting time (ACT): 73/30 seconds; fibrinogen: 1.17g/L). Platelets count was first normal but decreased rapidly and profoundly. All of the former laboratory results were aggravated within the next 24 hours. Symptomatic reanimation measures were initiated promptly: transfusion of 10 units of fresh frozen plasma (FFP), 4g of fibrinogen, 2g tranexamic acid, calcium supplementation, and the correction of the hyperkalemia, adequate vascular filling and diuretic administration. Trans-thoracic Cardiac echography showed evidence of pulmonary artery hypertension (PAH) at 80mmHg with a right ventricular dilated cavity. Fetal debris were present in the broncho-alveolar lavage fluid. Fetal debris include immature squamous cells.

**Figure 1:**
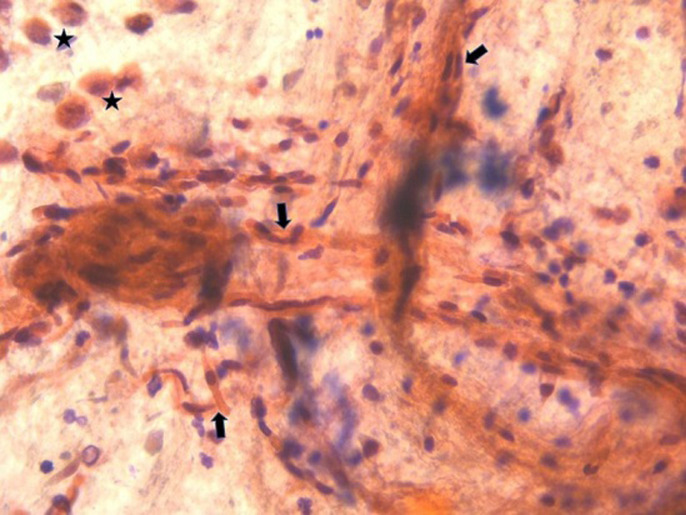
presence of immature squamous cells and macrophage in the broncho-alveolar lavage fluid (arrow: immature squamous cells; star: macrophage)

In our case, cytology showed only immature squamous cells (Figure 1). Based on all these clinical and para-clinical elements the diagnosis of AFE associated with disseminated intravascular coagulation (DIC) was established. The patient was intubated for 25 days; she required multiple session of renal hemodialysis for the acute renal failure. She developed ventilator-associated pneumonia (VAP) that was treated with wide spectrum antibiotherapy. The outcome was fortunately favorable and the patient was finally extubated and all biological elements went back to normal ranges.

## Discussion

Amniotic fluid embolism remains one of the most enigmatic and devastating condition in obstetrics due to its complex and poorly understood physiopathology and the potential gravity of its manifestations which can be lethal in some cases. This condition consist on a severe maternal reaction to amniotic fluid contents entering the circulation. Contemporary etiological hypotheses suggest that this syndrome results of, in addition to mechanical obstruction of pulmonary vessels, an abnormal activation of humoral and immunological mechanisms, and the release of vasoactive and procoagulant substances into the maternal circulation triggering an actual ‘anaphylactoid syndrome of pregnancy´ [4, 5]. Features of anaphylactic reaction were early observed in the case of our patient (cardiovascular collapse associated with a cutaneous rush) and our initial management was oriented towards an anaphylactoid shock secondary to the antibiotic administration. However, the condition actually was triggered little before the antibiotic administration announced by the postpartum bleeding. The clinical presentation of the AFE is unspecific with the wide spectrum of manifestations. Kanayama *et al*. suggested that AFE can be separated into two types: cardiopulmonary collapse type (classic type) and DIC type, with a relative prevalence of only one-third to the first and two-thirds for the second type [6].

In our case the onset of the condition was announced by the postpartum hemorrhage shortly followed by the cardiac collapse and the convulsive seize due to cerebral hypo-perfusion. In fact, the initial laboratory features, besides the cardiopulmonary collapse markers (metabolic acidosis and high lactates levels), noted elements of DIC (fibrinogen 1.17g/L; prothrombin: 37%; ACT 73/30 seconds; and a decreasing platelets count). These elements classes our case in the type two AFE with DIC. Therefore, we promptly initiated transfusion of FFP, platelets and fibrinogen to control the homeostasis and stop the bleeding. The diagnosis of AFE is based primarily on clinical observations related to the specific context of pregnancy. Detection of squamous cells or other debris in the pulmonary arterial bed of women is neither specific nor sensitive for the diagnosis of AFE [4, 7]. In fact, fetal debris include different components like immature squamous cells, fetal hair, or mucin...The identification of immature squamous cells is purely morphological. They are different from mature cells which can come from a possible contamination during endoscopy. The mature cells are larger and have a more abundant cytoplasm. Finding fetal squamous cells or other fetal debris in the broncho-alveolar lavage is encountered especially if the patient develop pulmonary oedema. The latter is responsible of an alveolar capillary membrane damage leading to the crossing of the amniotic fluid material from the maternal circulation to the pulmonary alveoli [7].

Furthermore, these debris were as well detected in a variety of critical illnesses including complicated preeclampsia, cardiac disease, and septic shock... [4]. Moreover, cases of AFE without any detected fetal squamous cells during the aspiration of pulmonary artery blood were reported to be of a great prevalence (50%) [8]. Therefore, the presence of fetal squamous cells in the pulmonary artery bed is considered suggestive but not diagnostic of AFE syndrome [9]. Many biomarkers have been proposed to be of benefit for the diagnosis of AFE like the C1 esterase inhibitor activity... [10]. However, there is no gold standard diagnostic marker for AFE and further work is needed to determine whether these markers provide adequate sensitivity and specificity for fatal and nonfatal AFE [6, 10].

In the case of our patient, due to the unavailability of biomarkers dosages in our laboratories, the diagnosis of AFE was based on a number of elements: the context of peri-partum, the association of cardio-respiratory collapse and a DIC, the signs of pulmonary arterial hypertension in echocardiography (PAH up to 80mmHg) and the presence of fetal debris in the bronchial aspiration.

## Conclusion

The upper is a description of a case of DIC-type AFE. The initial symptoms were uterine atony and bleeding secondary to DIC. The cardio-respiratory collapse leaded to a multi-visceral failure. Early diagnosis and prompt administration of blood products, including clotting factors and platelets, were essential for patient recovery.
